# Insights from establishing a high throughput viral diagnostic laboratory for SARS-CoV-2 RT-PCR testing facility: challenges and experiences

**DOI:** 10.3389/fpubh.2023.1122715

**Published:** 2023-04-17

**Authors:** Sanchita Roy Pradhan, M. H. Yashavarddhan, Ashish Gupta, Pramod Kumar, Anuj Kumar, Nazneen Arif, Usha Agrawal, R. Suresh Kumar, Shalini Singh

**Affiliations:** ^1^High-Throughput Viral Diagnostic Laboratory (HTVDL), ICMR - National Institute of Cancer Prevention and Research (NICPR), Ministry of Health and Family Welfare, Government of India, Noida, Uttar Pradesh, India; ^2^Department of Histopathology and Cytology, National Institute of Pathology, Safdarjung Hospital, New Delhi, India

**Keywords:** SARS CoV-2, coronavirus, COVID-19, viral diagnosis, RT-PCR

## Abstract

**Background**: The World Health Organization declared the coronavirus disease 2019 (COVID-19) a global pandemic on 11 March 2020. Identifying the infected people and isolating them was the only measure that was available to control the viral spread, as there were no standardized treatment interventions available. Various public health measures, including vaccination, have been implemented to control the spread of the virus worldwide. India, being a densely populated country, required laboratories in different zones of the country with the capacity to test a large number of samples and report test results at the earliest. The Indian Council of Medical Research (ICMR) took the lead role in developing policies, generating advisories, formulating guidelines, and establishing and approving testing centers for COVID-19 testing. With advisories of ICMR, the National Institute of Cancer Prevention and Research (NICPR) established a high-throughput viral diagnostic laboratory (HTVDL) for RT-PCR-based diagnosis of SARS-CoV-2 in April 2020. HTVDL was established during the first lockdown to serve the nation in developing and adopting rapid testing procedures and to expand the testing capacity using “Real-Time PCR.” The HTVDL provided its testing support to the national capital territory of Delhi and western Uttar Pradesh, with a testing capacity of 6000 tests per day. The experience of establishing a high-throughput laboratory with all standard operating procedures against varied challenges in a developing country such as India is explained in the current manuscript which will be useful globally to enhance the knowledge on establishing an HTVDL in pandemic or non-pandemic times.

## 1. Introduction

The coronavirus disease 2019 (COVID-19) was first identified in Wuhan city of China (1). In December 2019, the first outbreak of COVID-19 was reported in the Hubei Province of Wuhan with viral pneumonia in several patients which was later found to be associated epidemiologically with the Huanan seafood market in Wuhan (2). The first case of COVID-19 in India was reported on 27 January 2020 by a 20-year-old woman from Kerala who had a travel history to China (3, 4). Given the government's restrictions on air travel, it was observed that the main entry point of COVID-19 infection to India was by infected individuals who had traveled abroad (3, 4). On 24 March 2020, there were 564 confirmed cases of COVID-19 in India. A nationwide lockdown of 21 days was also declared to control the spread of COVID-19 cases (5). To identify the infected individuals within the general population and healthcare setup, along with lockdown rules, the adoption of extensive testing was required. On 3 April 2020, the ICMR National Institute of Cancer Prevention and Research, Noida entrusted the task of establishing a high-throughput viral diagnostic laboratory for testing samples of COVID-19 suspected patients using the RT-PCR test. A team of scientists along with technical, civil, electrical engineering, and administrative personnel worked together and established the HTVDL facility within 4 weeks.

The pathogen is considered in Risk Group 3 according to its transmission characteristics and genome analysis (6). The facility was planned to keep in mind the standard safety protocols required for the BSL 2 facility. A committee was constituted to plan the included civil electrical engineering works in an identified build-up area, laboratory workflow and biosafety, and requirements of consumables, equipment, and manpower. Self-installation of equipment such as biosafety cabinets, automated RNA extraction machines, real-time PCR, and other basic laboratory equipment was done by the scientist under the guidance of engineers of manufacturers using video calls when needed. Scientists and techno-managerial staff were hired to run the HTVDL facility 24 × 7. Standard operating procedures (SOPs) were developed, and the staff was trained accordingly. Quality assurance measures were implemented, and laboratory registration was done as per ICMR requirements. On 27 July 2020, the high-throughput viral diagnostic laboratory in Noida, along with other two laboratories in Kolkata and Mumbai, was inaugurated by the Honorable Prime Minister of India, who dedicated the laboratory to the service of the nation, with a capacity to perform 6000 RT-PCR tests per day to serve eight districts in Uttar Pradesh and few district hospitals in Delhi. Though the catchment area has ~90 million population, we could serve these regions with our capacity of 6,000 RT-PCR tests/day. This study describes the establishment of the laboratory that can be adopted for the diagnosis of contagious diseases such as COVID-19, as well as the experiences and challenges encountered during the establishment process. The terms HTVDL and HTL are used interchangeably throughout.

## 2. Context

### 2.1. Summary of challenges for the establishment of the high-throughput viral diagnostic laboratory for COVID-19 testing in ICMR-NICPR, Noida, UP

There were unprecedented challenges faced during the nationwide lockdown in setting up the laboratory that includes lack of manpower for civil and electrical work and renovation; disruption in the supply chain of materials required for construction due to the lockdown; recruitment of skilled staff for running the laboratory; and arranging for accommodation and the safety of the staff from infection. The Gautam Buddha Nagar District administration helped and facilitated the preparation of movement passes for laboratory construction workers and the restoration of the supply chain. Recruitment was done online through video conferencing. The accommodation and conveyance were facilitated by the Indian coast guard, and the dormitories were only for the working staff comprising three teams working 24 × 7. Biosafety measures were taken by developing standard operating procedures to ensure the safety of all staff and to prevent any cross-contamination in the laboratory processes.

### 2.2. Establishing the diagnostic real-time PCR laboratory: processes and challenges

#### 2.2.1. Laboratory layout and workstream of high-throughput viral diagnostic laboratory

**Figure 1:**
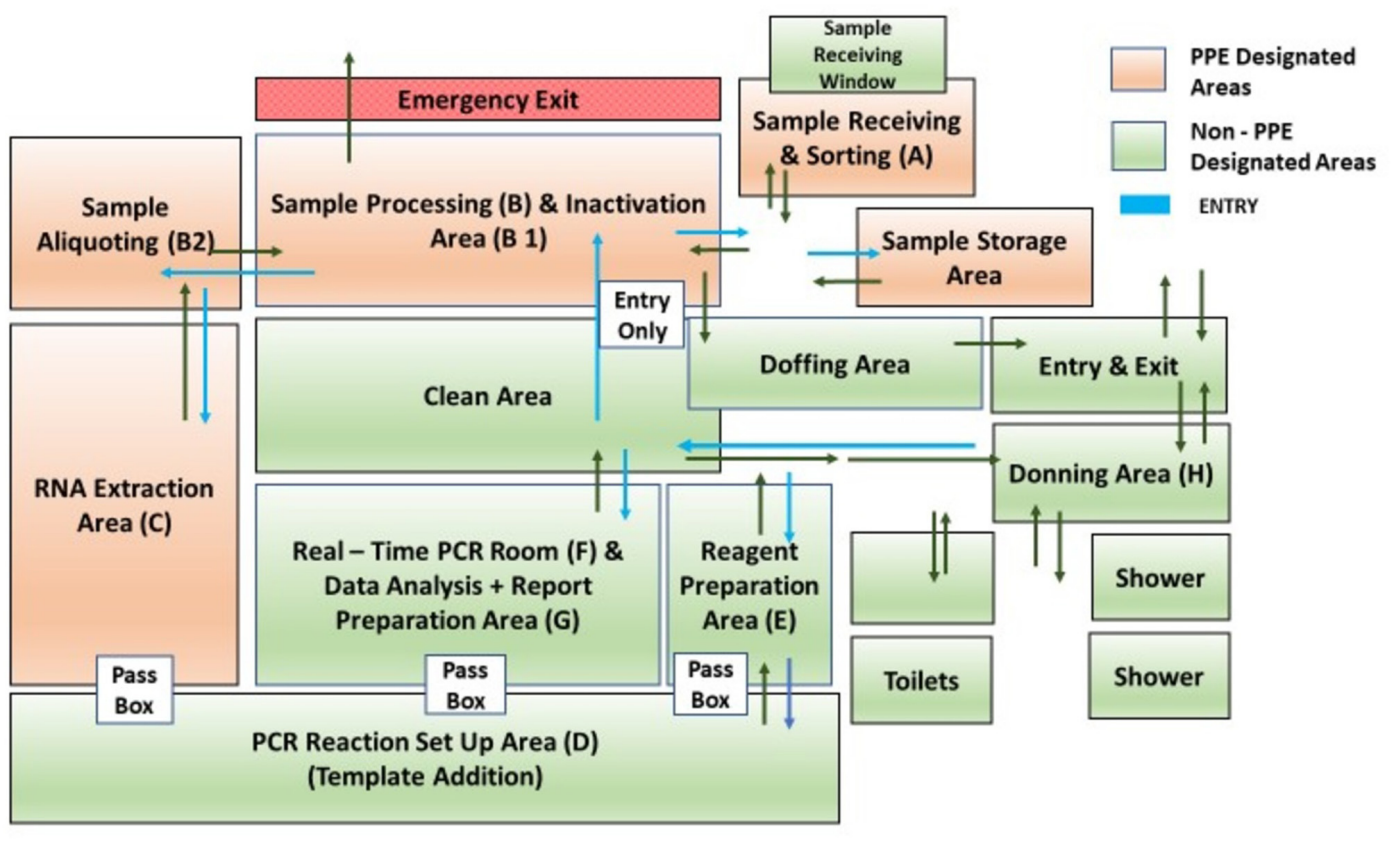
Detail laboratory layout of HTVDL-NICPR, Noida.

The HTVDL is a biosafety level 2 (BSL 2) laboratory with an installed capacity for 6,000 real-time PCR tests per day. With the available automated RNA extraction and real-time PCR facility, the maximum capability of the laboratory was decided to be 6,000 samples per day. The laboratory layout was planned to ensure that all the areas were cross-contamination free. The detailed layout of the facility is shown in Figure 1.

The facility has been divided into eight different sections (A–H) based on the potential for infection (Table 1).

**Table 1:** Potentially infected and non-infected or clean areas of HTVDL-NICPR, Noida.

**SL. No**.	**Potentially infected area**	**SL. No**.	**Non-infected/Clean area**
**A**	Sample Reception area	**D**	Real-Time PCR Reaction Set Up Area (RNA sample & PCR reagent mixing)
**B**	Sample Processing Area	**E**	Reagent Preparation Area
	**B1**. Sample Inactivation	**F**	Real-time PCR area
	**B2**. Sample Aliquoting Area	**G**	Facility for data analysis and report preparation
**C**	RNA Extraction Area	**H**	Donning area (PPE wearing)

The areas from A to C had been assigned as potentially infected areas, requiring the use of personal protective equipment (PPE), while the areas from D to H had been assigned as clean zones. The entry and exit points for staff working in clean and PPE areas were made separate to prevent cross-contamination.

The staff working in clean zones had been assigned different entry and exit points and crossing over of staff between these two zones was strictly prohibited.

Other areas in the adjacent rooms of the main laboratory included a control room for electrical control, a record room for maintaining records, a store room for storing laboratory consumables, a utility room for staff, an autoclaving area for the decontamination of laboratory wastes, restrooms, and a shower for convenience of staff.

## 3. Details of key programmatic elements

### 3.1. Standard operating procedures (SOPs) for different areas of the facility

Standard operating procedures were prepared for different functional areas of the laboratory. Training of all personnel engaged in the laboratory premises was done to assure minimal chances of contamination and promote safety and high-quality results.

**Figure 2:**
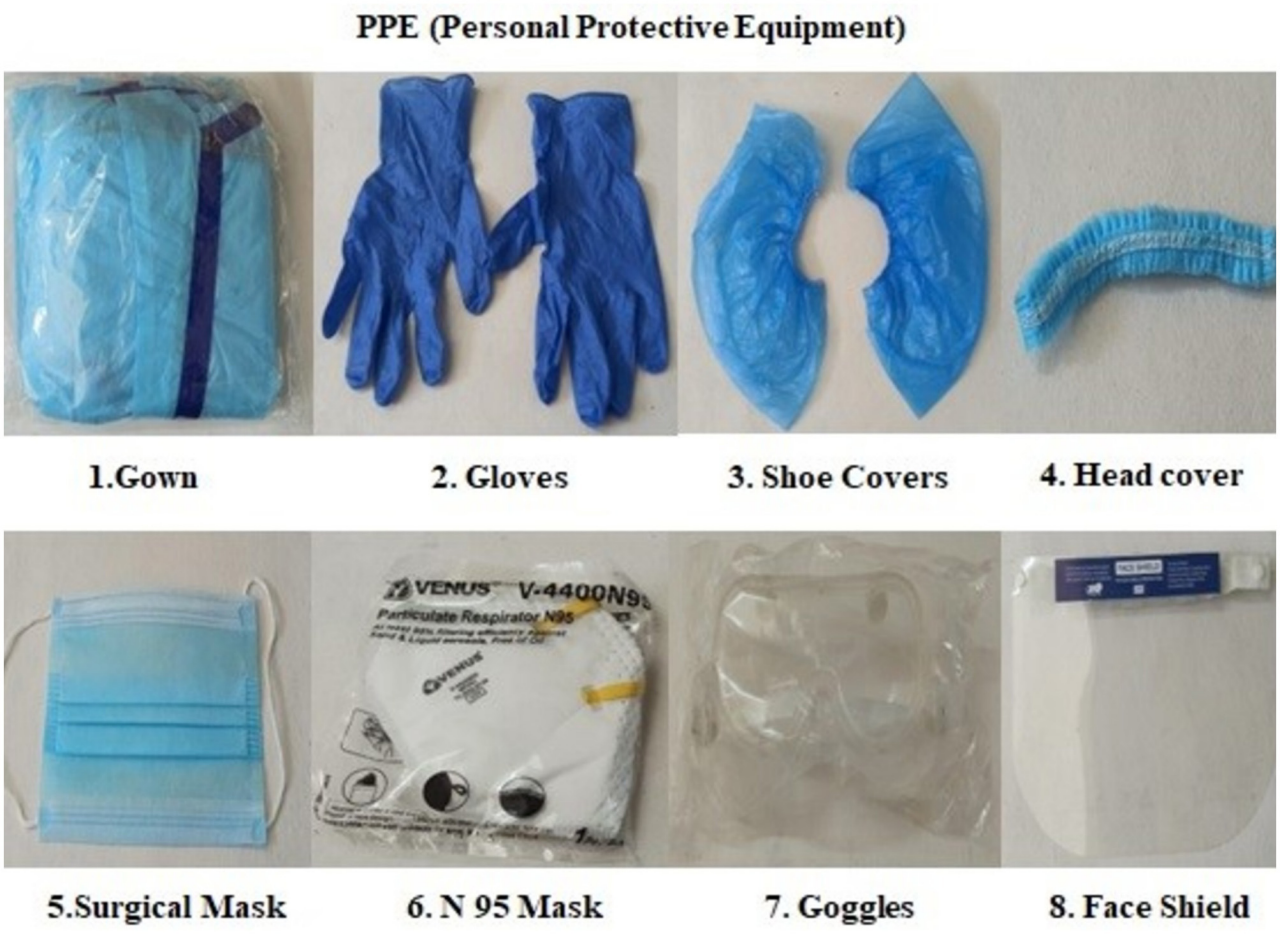
Personal protective equipment.

The staff working in potentially infected areas of the laboratory were required to wear PPE (Figure 2). Before entering the facility, all staff had to undergo hand hygiene protocols, as shown in Supplementary Figure 1.

### 3.2. Sample testing workflow

**Figure 3:**
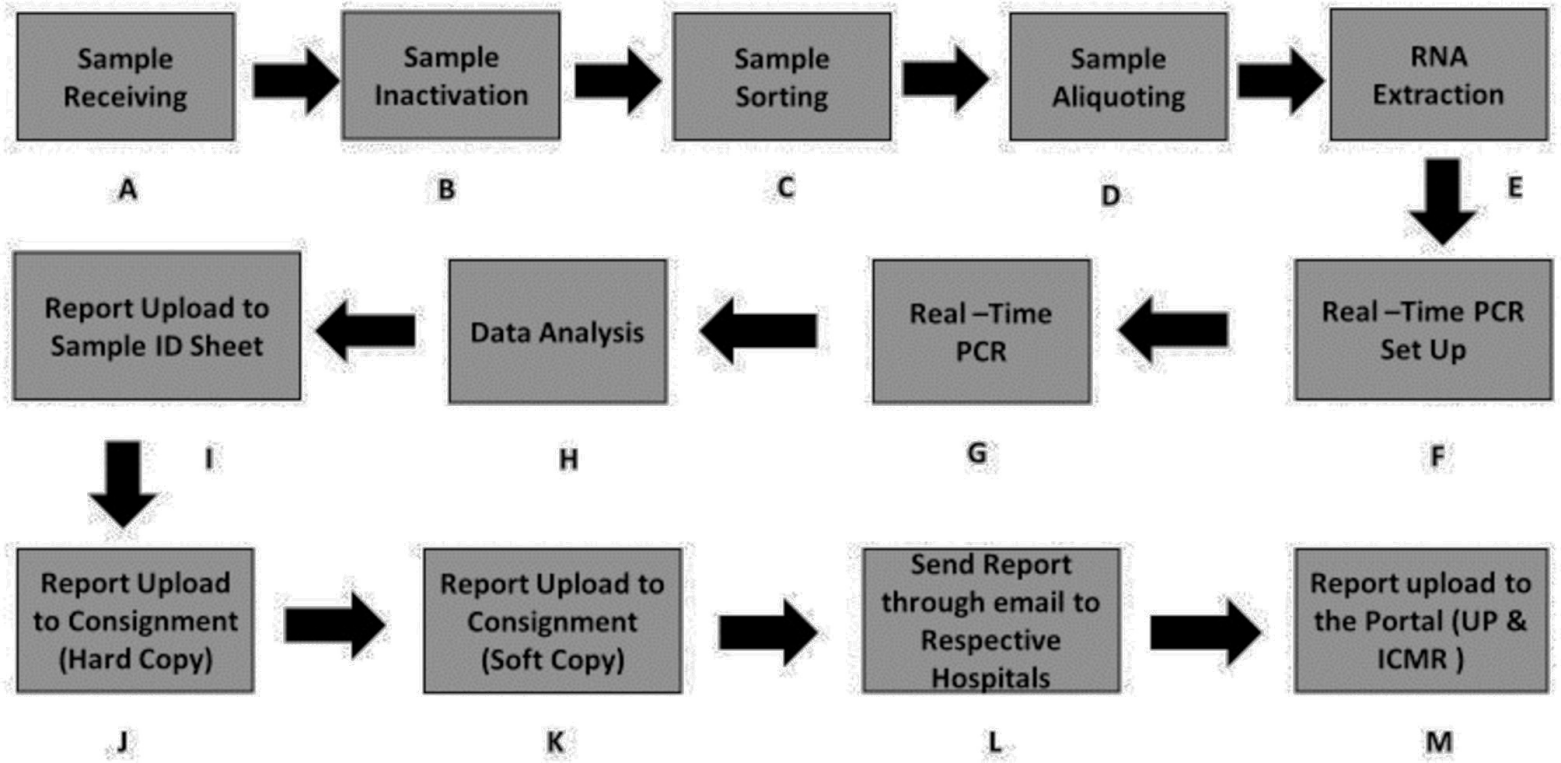
Workflow of sample processing from the sample receiving to reporting on the data portal. **(A)** Sample receiving. **(B)** Sample inactivation. **(C)** Sample sorting. **(D)** Sample aliquoting. **(E)** RNA extraction. **(F)** Real-time PCR Set UP. **(G)** Real-time PCR. **(H)** Data analysis/Data interpretation. **(I–M)** Steps to data upload on the portal.

The workflow of sample testing was designed in such a way that it consumes the least possible time for testing while reporting results in a timely and efficient manner using an automated/semi-automated approach. A reduced sample capacity during the initial operation of the testing facility helped to resolve challenges and technical issues. The workflow is represented diagrammatically in Figure 3.

#### 3.2.1. Sample receiving (A)

Samples from different districts of western UP and Delhi hospitals were received for testing at the sample receiving area (Supplementary Figure 2). The real challenge was tracking the submitted samples because samples from all centers were received at the same time and were assigned unique specimen referral form (SRF) IDs. The HTVDL assigns a specific number code to each sample before processing it. Hence, to ease the identification, meticulous recording of samples received begins at the security gate of the campus, where the details of hospitals/districts, the number of samples, and the time of arrival were noted. These entries were always corroborated with testing reports sent for data entry at a unified, uniform countrywide software platform designed by the ICMR to upload all test results (ICMR portal: https://cvstatus.icmr.gov.in/) and access results (https://report.icmr.org.in). This makes it easy to visualize state-wise, countrywide infection rates at a single platform by healthcare administrators. Missing samples were coded as sample not received (SNR), and samples with leakage or wrong labeling were identified and separated for the record.

#### 3.2.2. Sample inactivation (B)

A sample ID sheet was prepared by HTL, which depicts the 96-well plate format to be followed throughout the test procedures (Supplementary Figure 8). The sample ID sheets were cross-checked and signed by a designated scientist every time to ensure the integrity of the data.

#### 3.2.3. Sorting (C)

After verifying the samples received, the sample containing VTM tubes was incubated in a hot air oven at 56°C for 30 min for heat inactivation. After heat inactivation, samples were sorted, as per the line-listing provided by the hospitals, and a NICPR-HTL's laboratory ID is provided to each sample on a day-to-day basis (Supplementary Figures 3–6). Each sample received was assigned a unique ID.

#### 3.2.4. Sample aliquoting (D)

After successful heat inactivation at 56°C for 30 min in a hot air oven, 200 μl of the sample was aliquoted in deep well plates (Supplementary Figure 7). Out of 96 wells, 94 wells were aliquoted with samples and two wells were left for positive and negative control. The aliquoting process was carried out in the BSL 2 cabinet. A single sample per well was aliquoted and tested during the pandemic time. However, when the positivity rate decreases to <5% in a cohort/specific district, the pooling of samples was adopted as per ICMR guidelines, i.e., an equal volume of five (when the infective rate is <5%) or 10 samples (when the infective rate is <1%) were mixed up together, and 200 ul of the sample mix was taken for the testing process. The pooling method helped to decrease the turnaround time and cost per test, i.e., reducing the amount of the consumables and reagents used and also shortening the reporting time (Pooling guidelines: https://www.icmr.gov.in/pdf/covid/strategy/Advisory_on_feasibility_of_sample_pooling.pdf).

The same sample ID sheet along with samples was transferred from aliquoting area to RNA extraction area, and passed on to next template addition area with isolated RNAs. During the process, critical cross-checking is carried out before proceeding to the next section.

#### 3.2.5. RNA extraction (E)

The total RNA was extracted with an automated RNA extraction system (MGISP960), which has the maximum capacity of testing 192 samples in a single run (Supplementary Figure 9). A single run of RNA extraction of 96 samples took 55 min. Decontamination took ~20 min for pre- and post-procedures. The working platforms of the automated RNA extraction system were cleaned thoroughly using the RNase AWAY and 70% ethanol after each RNA extraction run. The pre-cleaning was done before running the first batch of the day to ensure contamination-free RNA extraction of the samples (Supplementary Figure 10).

Challenges in this area were that the prepared reagents for RNA extraction need to be utilized within 30 min of preparation. Another challenge was that there were chances of volume variations in various wells, which needed to be monitored every time manually. Therefore, time management and monitoring of every batch of extraction were critical for a high-capacity laboratory such as ours.

#### 3.2.6. PCR reaction set-up area (F)

In the real-time PCR setup area, extracted RNA (5–10 μl) was added to the freshly prepared reagent plate as per the kit protocol. The procedure was followed in separate exclusive BSL 2 cabinets designated for reagent preparation and RNA addition.

Proper handling of extracted RNA was important to maintain contamination-free manual template addition that reduces the number of unamplified samples.

**Figure 4:**
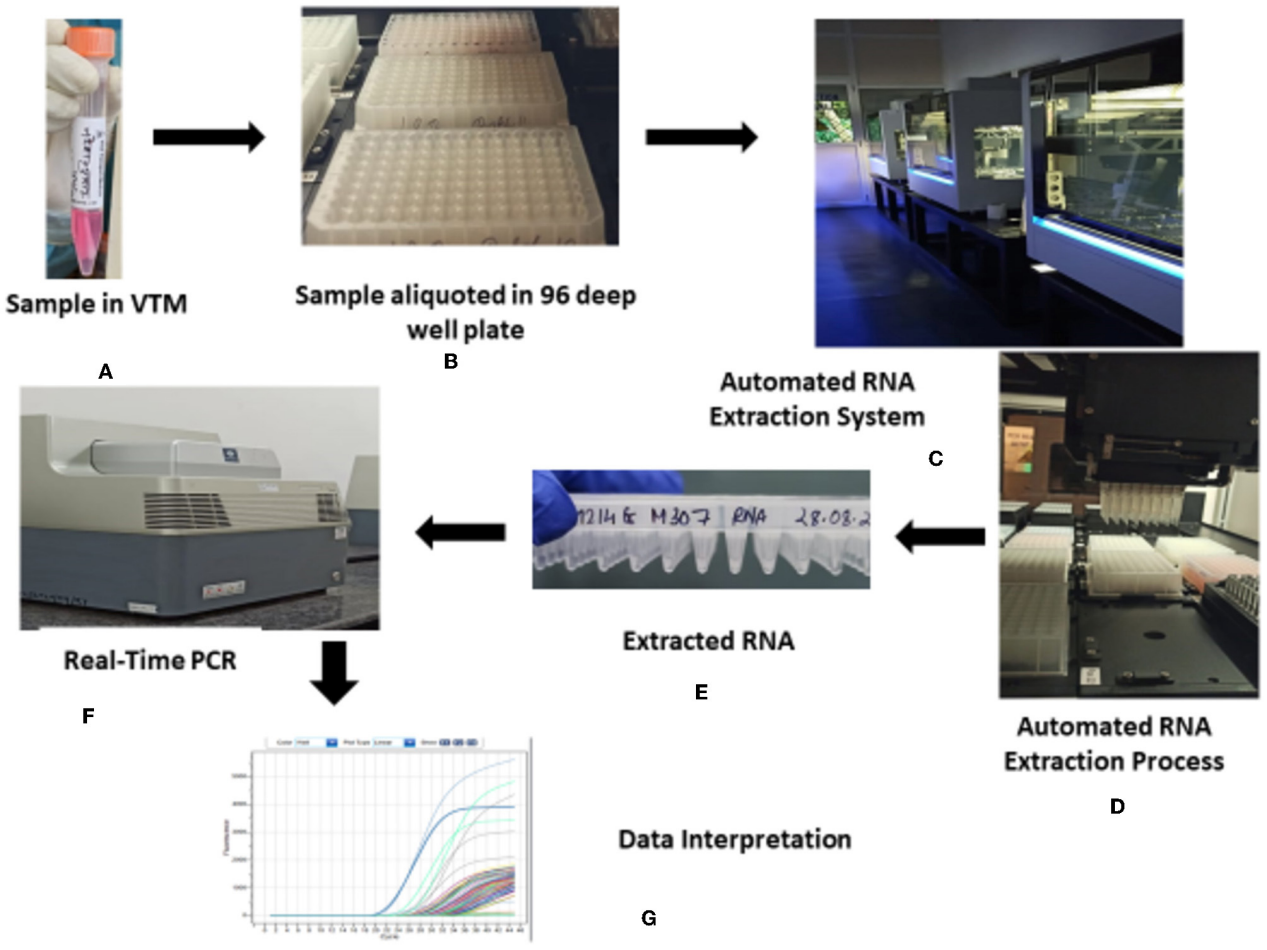
Overview of the HTL, NICPR COVID-19 testing consortium. **(A)** Sample in VTM. **(B)** Sample aliquoted in 96 deep well plate. **(C)** Automated RNA extraction system. **(D)** Automated RNA extraction process. **(E)** Extracted RNA. **(F)** Real-time PCR. **(G)** Data interpretation.

The overview of the COVID-19 testing consortium is depicted in Figure 4. Proper cleaning of the biosafety cabinets was done before and after the template addition. In addition, chemical fumigation was done on a regular basis to minimize the chances of contamination.

#### 3.2.7. Real-time PCR (G)

COVID-19 was confirmed in samples using the real-time fluorescent RT-PCR kit. The RT-PCR kits used for COVID-19 detection were the Taqman probe-based single-step real-time PCR kits. The RT-PCR kits were selected to amplify two or multiple viral gene targets such as ORF1ab and N gene with RNAse P as an internal control. The RT-PCR kits with fluorophore probes that were compatible with HTL RT-PCR machine filters, i.e., FAM, HEX, and ROX were used (Supplementary Figure 11). The probes were oligonucleotides with a reporter fluorophore attached at the 5' end and a quencher attached at the 3' end. Specific primers and probes were also developed as an internal reference, with fluorophores VIC/HEX attached at the 5' end as reporters.

The reaction was set up with 5–10 μl of RNA samples and the master mix to individual wells of the plate. RT-PCR steps typically contain reverse transcription at 50°C for 15–30 min, followed by a standard PCR procedure, which includes denaturation at 94°C for 5 min and 94°C for 15 s, followed by annealing and extension at 60°C. Once strands get separated, the probes get attached to the target. During extension, the Taq polymerase cleaves the reporter, relieving the free dye for detection.

This cleavage results in the generation of fluorescent signals by the reporter dye, and amplification represents signal intensity. Monitoring the fluorescence intensities in real-time allowed us the qualitative/semi-quantitative detection of desired nucleic acid in specimens.

#### 3.2.8. Data interpretation (H)

**Figure 5:**
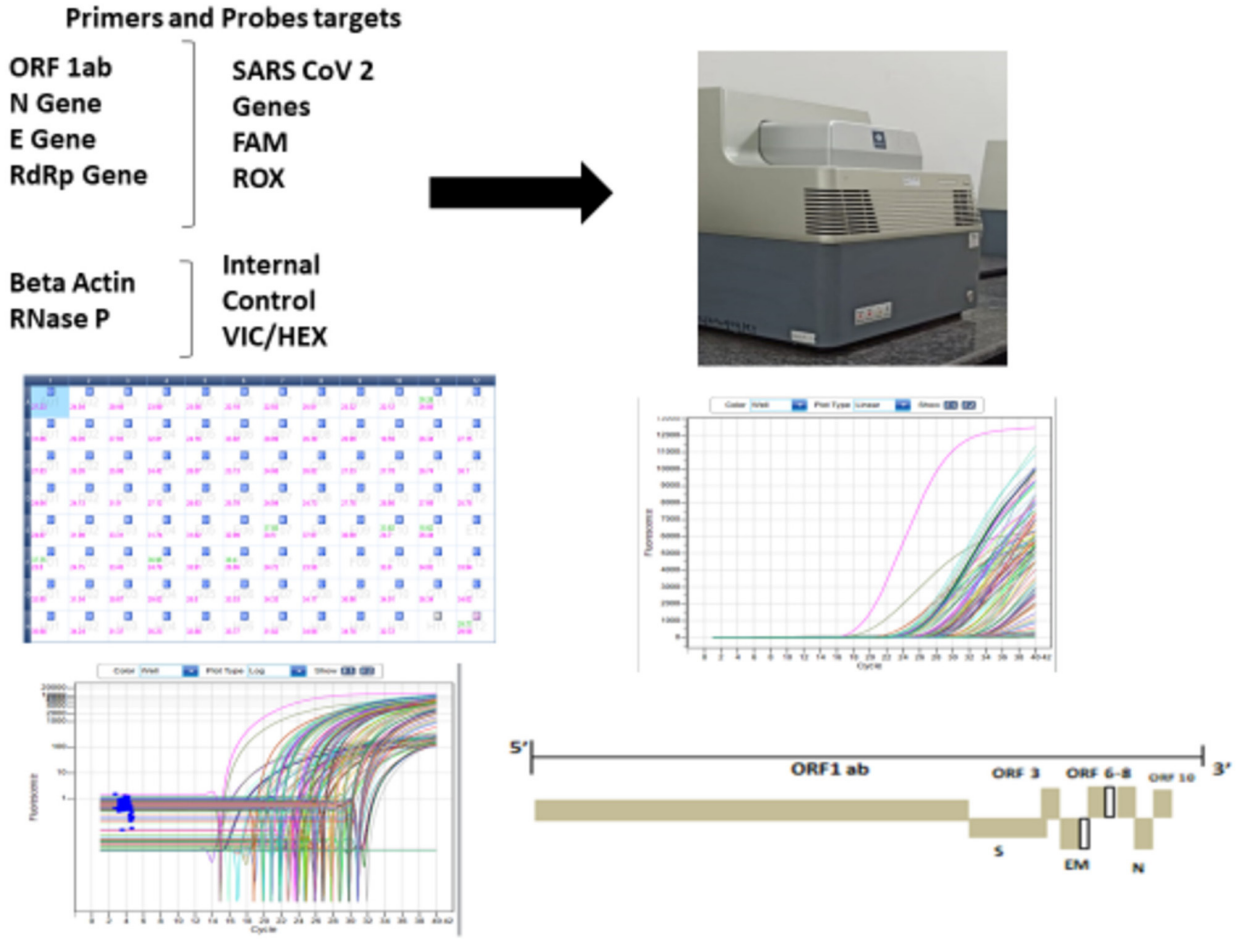
Workflow of real-time PCR and data interpretation.

Data interpretation of the Ct values was done after each real-time PCR run of samples received (Figure 5). The lower Ct value denotes higher copy numbers, and the higher Ct value denotes lesser copy numbers of viral copies in the sample (7). The specimen was considered positive if the standard curve of the target gene (ORF/RdRp/ Ngene) was S-shaped with a Ct value not higher than 38–40 as per manufacturer's instructions or else reported negative. However, the internal control value needs to be within the range along with the S-shaped curve to report as confirmed negative. The cutoff Ct value is to be considered positive (up to 35–38 Ct) or negative (>35–38) and was followed as per the advisory of the Indian Council of Medical Research.

#### 3.2.9. Data upload on the portal (I to M)

The sample was retested if there was no value or presence of a higher Ct value than the Ct value specified for the internal control. The Ct values thus generated after each real-time PCR run were documented in the sample ID sheets with the laboratory ID numbers of the samples tested. The same data were entered on the hard copies of the line list and also in the soft copy, and the soft copies were emailed to the respective hospitals, and the same results were uploaded on the COVID-19 portal of the ICMR and Uttar Pradesh state. An example of portal entry on the incidence of positive cases in Amroha and Moradabad has been portrayed (till June 21) as the graphs in Supplementary Figure 12 and for Delhi in Supplementary Figure 13.

## 4. Discussion

### 4.1. Challenges and quality control measures of SARS-CoV-2 testing

Considering the various technologies available, the RT-PCR test has been the preferred technique as it confirms sensitivity, specificity, and accuracy and can be adopted in a high-throughput format with limited time requirements (8). Various RNA gene targets such as envelope (env), nucleocapsid (N), spike (S), RNA-dependent RNA polymerase (RdRp), and the first open reading frame (ORF1) genes were used in this assay (9). Most of the commercially available and *in vitro* diagnostics-approved kits have multiple targets along with one internal control in a single reaction which increases the accuracy of this assay. However, it was essential that laboratories should adopt good quality control (QC) practices to establish confidence in the reliability of the test. This part of the manuscript highlights the challenges faced and enlightens best practices for good quality control as follows.

The best practices for quality control that helped in achieving 100% specificity of the test were followed in all sections of the laboratory.

#### 4.1.1. Sample collection, storage, and inactivation

Nasopharyngeal and oropharyngeal swab samples were instructed to be collected in viral transport medium (VTM) vials from the collection center and transported to the viral diagnostic laboratory. The VTM helped in maintaining the quality and stability of samples. Furthermore, good quality and lint-free swabs used at the time of collection prevent the blockage of the filter pipette tips by the lint if present, which would affect the quantity of required sample volume for RNA extraction and in turn affect the PCR results. A dry and cold environment was ideal to store and transport the VTM vials to maintain cell stability.

For safety precautionary measures, the samples were inactivated at the proper temperature (56°C) and incubation time (30 min). Very high temperatures or longer incubation may degrade the nuclear material.

#### 4.1.2. Aliquoting

During the aliquoting process, the following things were monitored.

The sample unique ID is written on the sample ID sheet.

The arrangement of samples in sequential order.

Ensuring proper distribution of sedimented cells, the sample was pipetted or gently mixed by vertexing while aliquoting.

#### 4.1.3. Isolation of RNA

An IVD-approved RNA extraction kit was used for the viral nucleic acid extraction, and the manufacturer's protocol needs to be followed for good-quality purified RNA. The samples were mixed properly before carrying out the RNA extraction procedure.

**Table:** Record sheet for MGISP960 automated RNA extraction system (ARES).

Date	Name of ARES operators	Number of samples tested	Sample receiving time (from aliquoting area)	RNA handed over to RT-PCR set-up area	Post clean (Y/N)	Signature

**Table:** Record sheet for L&B preparation.

Date	Number of reaction	L&B preparation time	L&B used by (Time)	Prepared by	Signature

The RNA extraction was performed using the magnetic bead-based method (automated extraction system). Reagent separation, lysis and binding (L&B), buffer preparation, and aliquoting in a 96-deep well plate format were simultaneously performed before starting the RNA extraction work. Reagent separation was done by a machine, which was kept in particular places, and the desired reagent volume was aliquoted as per script. The same could be done manually as well. Once the L&B buffer was prepared, it had to be used within 30 min, so the timing of preparation was also noted in the registers.

Moreover, registers for consumables used on a daily basis were made to track down the number of consumables used, which further helped to manage the inventory better. Maintenance of the consumable register also helped in generating data on store management, assessing consumable availability, and adopting various strategies as per the situation.

#### 4.1.4. Selection of PCR kit and PCR mix preparation

The RT-PCR-based COVID-19 test is regarded as the gold standard in molecular diagnosis. Though the kit is procured, we need to take precautions while selecting the kit. The ORF1 ab gene is reported as the most specific confirmation target but a less sensitive target, which may lead to false-negative results in asymptomatic patients or patients with low viral load. In addition, the mutability of target genes also gives false-positive results. Therefore, it is wise to select two or multiple targets at the time of kit selection, i.e., ORF1ab along with N gene or E gene or S gene needed to be targeted along with internal control, housekeeping gene, e.g., beta-actin and RNase P. Internal control plays an important role in the PCR-based test that shows the quality of the extracted RNA. The Ct value of internal control within the prescribed range indicates the quality of sample collection, RNA extraction, or re-extraction.

In case of re-extraction, if similar kinds of results were observed, then repeated sampling was needed.

Diagnostic kits with ultra-high detection sensitivity can detect the lowest copy numbers of target genes. This can be assessed by the serial dilution method by taking known positive samples. The lower limit of detection can be assessed by diluting the positives with 10-fold serial dilution. The kit with the highest detection capacity should be considered for testing purposes. As per the theoretical calculation, a 3.25 Ct difference for 10-fold dilutions should be considered the best result with no PCR inhibitors in any of the premix PCR solutions (10). A serial dilution of samples was performed, and a representative result is shown in Supplementary Figure 14.

Primer-probe or reaction mixture concentration is one of the important factors to avoid false-positive or false-negative results. A 500-nM concentration of primer and a 250-nM probe per sample were considered to give better results. As per the manufacturer's protocol or standardized protocol, the master mix was prepared and dispensed equally in a PCR tube or plate.

#### 4.1.5. Sample preparation and PCR setup

All the reagents were thawed completely and mixed properly before adding RNA. The exact volume of RNA template addition and mixing with mater mix was ensured. The tubes/plates were spun down without any bubbles. The presence of an air bubble in the mixture (Supplementary Figure 15), improper plate sealing (Supplementary Figure 16), or tube capping results in false or no amplification. Pipetting is another issue, and multichannel pipettes are generally used due to technical or handling issues.

The false-positive or false-negative results were due to poor specimen quality, incorrect sample collection, transportation, or laboratory processing procedures, and technological limitations. To counteract these, the operators were trained well in advance on the procedures and its limitation in performance to prevent any human errors.

#### 4.1.6. Result analysis

Data analysis is one of the major factors in predicting the raw data results. Most of the new generation multiplexing real-time PCR machines calculate the background fluorescence and set the baseline automatically. However, one must know that the theoretical considerations for setting up the baseline were a baseline value of 10 standard deviations or 1/10 of the (mean)maximum fluorescence unit. However, this needs to be checked for individual targets; if some of the curves were non-sigmoid, then the manual setting of the baseline was performed as shown in Supplementary Figure 17. The samples with a low viral load might show a non-sigmoidal curve due to a high Ct value. However, in such cases, the logarithm plots were observed to check the sigmoid shape of the curve (Supplementary Figure 18).

#### 4.1.7. Quality control assay

For optimal results, it is recommended to carry out quality control (QC) procedures every day. In this assay, quantitative tests for three controls need to be performed which include a negative control, a low-positive control, and a high-positive control. If the values of these controls were not in range, then reagent preparation and template addition were checked. After performing the same, if QC showed the result out of range, then PCR calibration was performed.

**Table:** Record sheet for consumables used.

Date	Number of samples tested	No. of robotic tips used	No. of deep well plate used	No. of PCR plate used	No. of plate sealer used	Signature

Similarly, one negative template control and one known positive control were provided with each batch of QC, along with the kit used to run the assay. If this value was not satisfactory, the batch was repeated.

### 4.2. Record keeping

The technical workflow of the high-throughput diagnostic laboratory was supported by our manual record-keeping system to track the samples received and tested through our facility. The hard copies filled with results were managed in files, date- and district-wise, and maintained in the record room, and soft copies with results were stored in a computer in the PCR data analysis areas. This is one of the laboratory management procedures for our checks. These records will not be accessible to healthcare workers.

### 4.3. Store management

In the nationwide lockdown scenario, inventory management was one of the biggest challenges faced by our facility. The stock and indent issued were maintained and tallied on a daily basis to manage the daily store availability of consumables.

The update on the consumables used in the testing has been maintained regularly to counter issues related to shortage. Stock registers had been used for recordkeeping of individual items used day-wise.

### 4.4. Bio-medical waste management

The high-throughput laboratory (HTL) at NOIDA, which has been set as a model at its best in following the best practices of biomedical waste (BMW) management, has been following the COVID-19 testing procedures since July 2020. All guidelines by the Ministry of Environment, India for BMW, have strictly been adhered to and followed for the collection and segregation of all types of waste. The HTL has kept separate color-coded bins in different areas of the laboratory to segregate the wastes according to their nature.

Double-layered biohazard bags are used for the collection of waste from the sample receiving area, BSL 2 facility, and PPE-doffing area to ensure proper containment and no leakage from sample vials. The various categories of BMW are to be segregated into different color-coded bins (Supplementary Figure 19).

Before autoclaving, bags were disinfected with 1% sodium hypochlorite dissolved in water and properly labeled as COVID-19 waste. Personal protective equipment (PPE), VTM vials, and plastic wares used in aliquoting were segregated in red bags and autoclaved according to standard operating procedures (Supplementary Figure 20). The autoclaved materials were transported to separate areas with protective enclosures for further disposal.

A common biomedical waste treatment and disposal facility (CBMWTF) was hired for the treatment and disposal of waste generated at the HTVDL. After all of the waste had been sanitized, HTL-trained staff handed over the waste to the common biomedical waste treatment and disposal facility (CBMWTF), which then loaded it onto vehicles to be taken for further processing (Supplementary Figure 21).

In HTVDL, an average of 70 kg/day of waste was disposed, with an appropriate log maintained for all biomedical wastes.

## 5. Recommendation

Direct connection of data and records from the laboratory information system with national or international public health surveillance systems proved to be crucial in monitoring the COVID-19 outbreak globally. The past SARS-CoV-2 outbreak and its unprecedented nature of spread have shown the world the significance of laboratory diagnosis of COVID-19 leading to better management not only for patients with a serious infection but also to limit the spread of the infection. To achieve better management of COVID-19, routine diagnostic testing needs to take place in a timely manner.

The challenges faced while establishing the high-throughput viral diagnostic laboratory were formidable. However, the experiences gained so far show that with perseverance and teamwork, those challenges can be overcome. The shared experiences will be of great knowledge source for establishing such a laboratory. We can control the global pandemic if institutes with molecular biology departments and resources come forward and replicate and amplify on the findings of this study. Furthermore, if there is constant support from government entities through funding to the testing facilities, it will enable uninterrupted services to tackle this global crisis and come up with better public health policies.

## Data availability statement

The original contributions presented in the study are included in the article/Supplementary material, further inquiries can be directed to the corresponding authors.

## Ethics statement

Written informed consent was obtained from the individual(s) for the publication of any potentially identifiable images or data included in this article.

## Author contributions

RSK and SS designed, partly performed, and wrote the paper content. SRP, MHY, and AG wrote and performed the experiments in RNA, RTPCR, and reagent setup, and waste management respectively. PK, AK, NA, and UA contributed to a lab setting up process. All authors contributed to the article and approved the submitted version.
