# Supplementary material for: Insights from establishing a high throughput viral diagnostic laboratory for SARS-CoV-2 RT-PCR testing facility: challenges and experiences
Source: Front Public Health. 2023 Apr 17;11:1122715. doi: 10.3389/fpubh.2023.1122715 (PMC10152062; doi:10.3389/fpubh.2023.1122715)
Supplement: Supplementary file 1 [file Data_Sheet_1.PDF]

Supplementary Material

Insights from Establishing a High throughput Viral Diagnostic Laboratory for SARS CoV-RT-PCR testing facility: Challenges and Experiences

Sanchita Roy Pradhan[1], M H Yashavarddhan[1], Ashish Gupta[1], Pramod Kumar[1], Anuj Kumar[1], Nazneen Arif[1], Usha Agrawal[2], R Suresh Kumar[1], Shalini Singh[1]

*** Correspondence:**

Dr. Shalini Singh : shalinisingh.icmr@gmail.com

Dr. R. Suresh Kumar: drramsuresh74@yahoo.com, suresh.kr73@gov.in

2. Supplementary Figures and Tables

2.1. Supplementary Figures

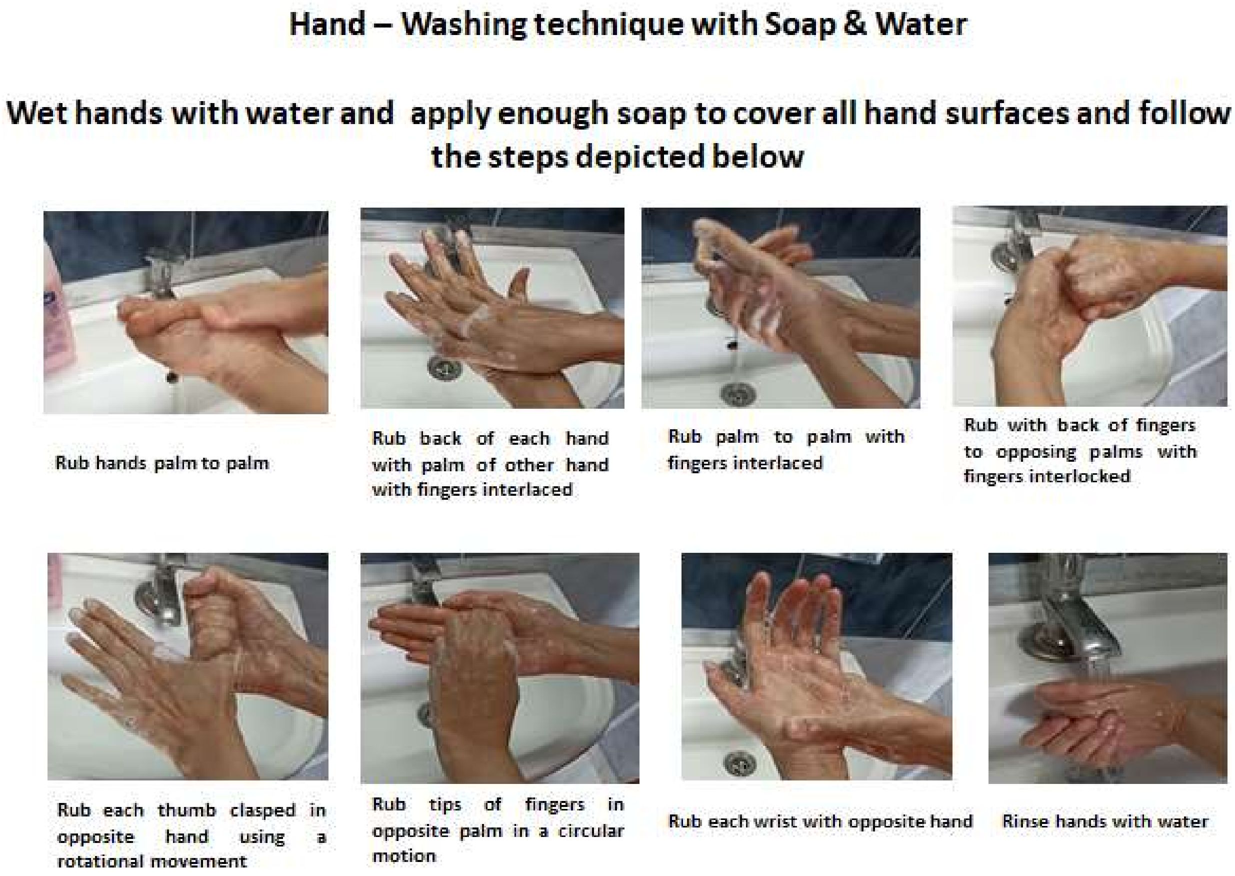

Figure 1: Hand washing before entry in to facility

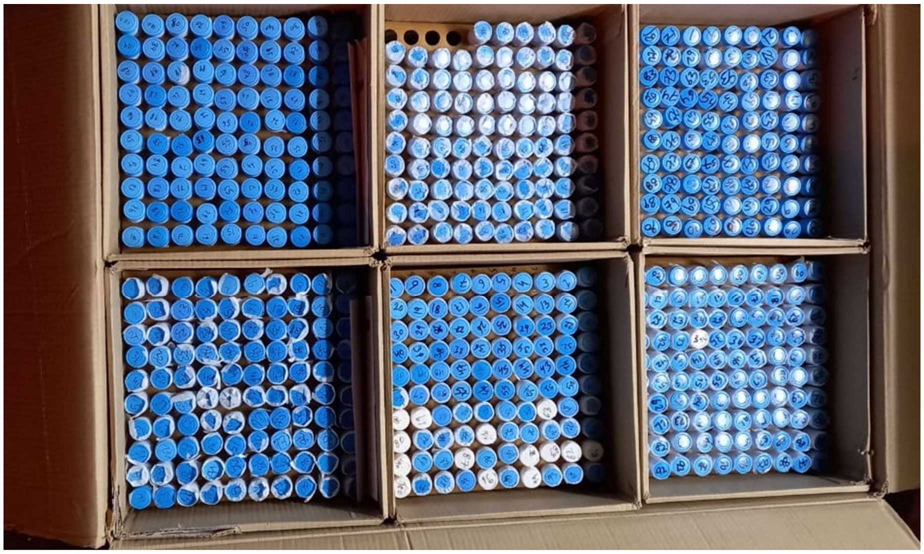

Figure 2: Samples received from different hospital

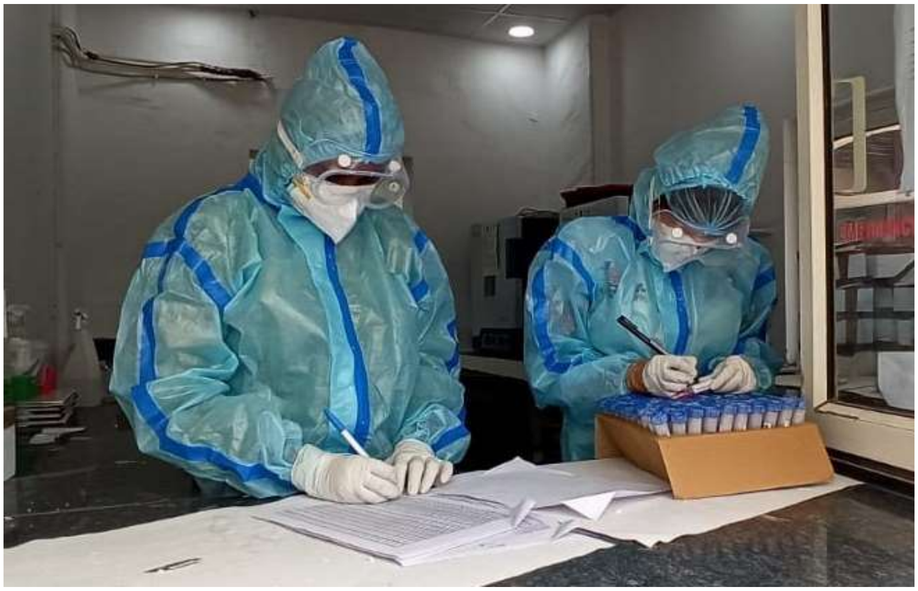

Figure 3: Staff sorting the samples

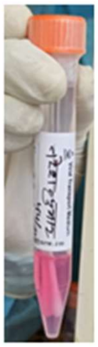

Figure 4

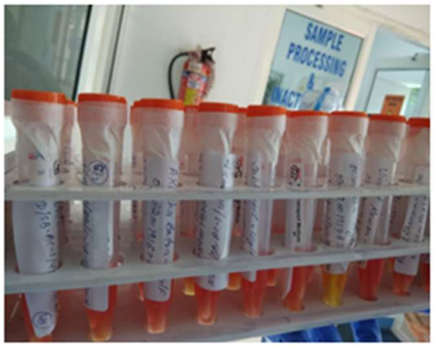

Figure 5

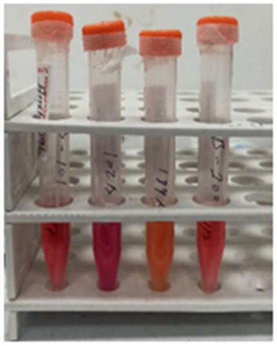

Figure 6

Figure 4,5 & 6: Before and after sorting

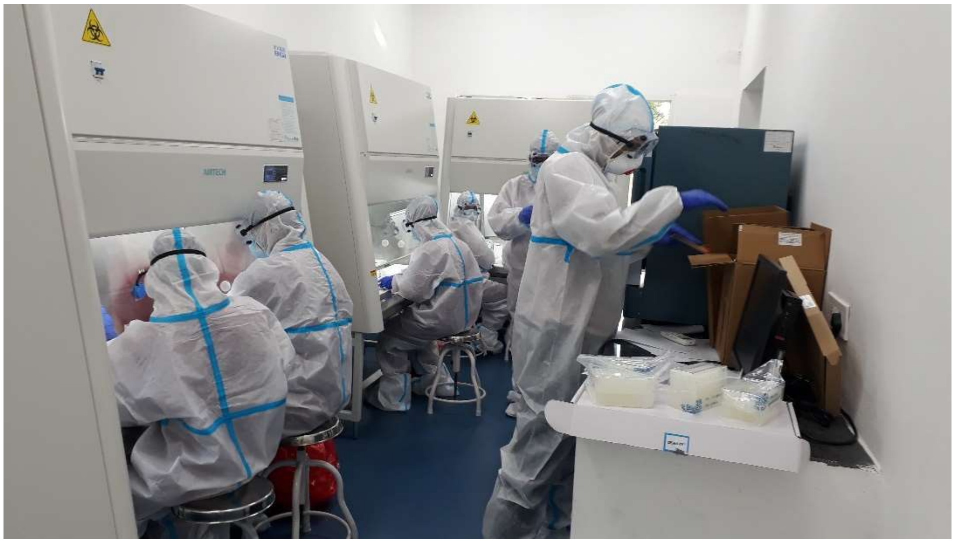

Figure 7: Staffs during sample aliquoting

Batch No. ……… Sample No. ……………… Aliquoting Date……….Sorting Date…………. Receiving date…..

	1	2	3	4	5	6	7	8	9	10	11	12
A	ID…… Results (Ct)	ID…… Results (Ct)	ID…… Results (Ct)	ID…… Results (Ct)	ID…… Results (Ct)	ID…… Results (Ct)	ID…… Results (Ct)	ID…… Results (Ct)	ID…… Results (Ct)	ID…… Results (Ct)	ID…… Results (Ct)	ID…… Results (Ct)
B	ID…… Results (Ct)	ID…… Results (Ct)	ID…… Results (Ct)	ID…… Results (Ct)	ID…… Results (Ct)	ID…… Results (Ct)	ID…… Results (Ct)	ID…… Results (Ct)	ID…… Results (Ct)	ID…… Results (Ct)	ID…… Results (Ct)	ID…… Results (Ct)
C	ID…… Results (Ct)	ID…… Results (Ct)	ID…… Results (Ct)	ID…… Results (Ct)	ID…… Results (Ct)	ID…… Results (Ct)	ID…… Results (Ct)	ID…… Results (Ct)	ID…… Results (Ct)	ID…… Results (Ct)	ID…… Results (Ct)	ID…… Results (Ct)
D	ID…… Results (Ct)	ID…… Results (Ct)	ID…… Results (Ct)	ID…… Results (Ct)	ID…… Results (Ct)	ID…… Results (Ct)	ID…… Results (Ct)	ID…… Results (Ct)	ID…… Results (Ct)	ID…… Results (Ct)	ID…… Results (Ct)	ID…… Results (Ct)
E	ID…… Results (Ct)	ID…… Results (Ct)	ID…… Results (Ct)	ID…… Results (Ct)	ID…… Results (Ct)	ID…… Results (Ct)	ID…… Results (Ct)	ID…… Results (Ct)	ID…… Results (Ct)	ID…… Results (Ct)	ID…… Results (Ct)	ID…… Results (Ct)
F	ID…… Results (Ct)	ID…… Results (Ct)	ID…… Results (Ct)	ID…… Results (Ct)	ID…… Results (Ct)	ID…… Results (Ct)	ID…… Results (Ct)	ID…… Results (Ct)	ID…… Results (Ct)	ID…… Results (Ct)	ID…… Results (Ct)	ID…… Results (Ct)
G	ID…… Results (Ct)	ID…… Results (Ct)	ID…… Results (Ct)	ID…… Results (Ct)	ID…… Results (Ct)	ID…… Results (Ct)	ID…… Results (Ct)	ID…… Results (Ct)	ID…… Results (Ct)	ID…… Results (Ct)	ID…… Results (Ct)	ID…… Results (Ct)
H	ID…… Results (Ct)	ID…… Results (Ct)	ID…… Results (Ct)	ID…… Results (Ct)	ID…… Results (Ct)	ID…… Results (Ct)	ID…… Results (Ct)	ID…… Results (Ct)	ID…… Results (Ct)	ID…… Results (Ct)	ID…… Results (Ct)	ID…… Results (Ct)

Aliquoted by: ……………………

Automated RNA extraction system
Name:……………………
Machine No ……………………
Signature……………………

Template Added by :……………

q-PCR Machine operator
Name:……………………
PCR Machine & Laptop/PC No……………
Signature……………………

Reviewed By……………………

Remarks……………………

Figure 8: Blank Sample ID sheet to fill the Real-Time PCR results and all relevant information

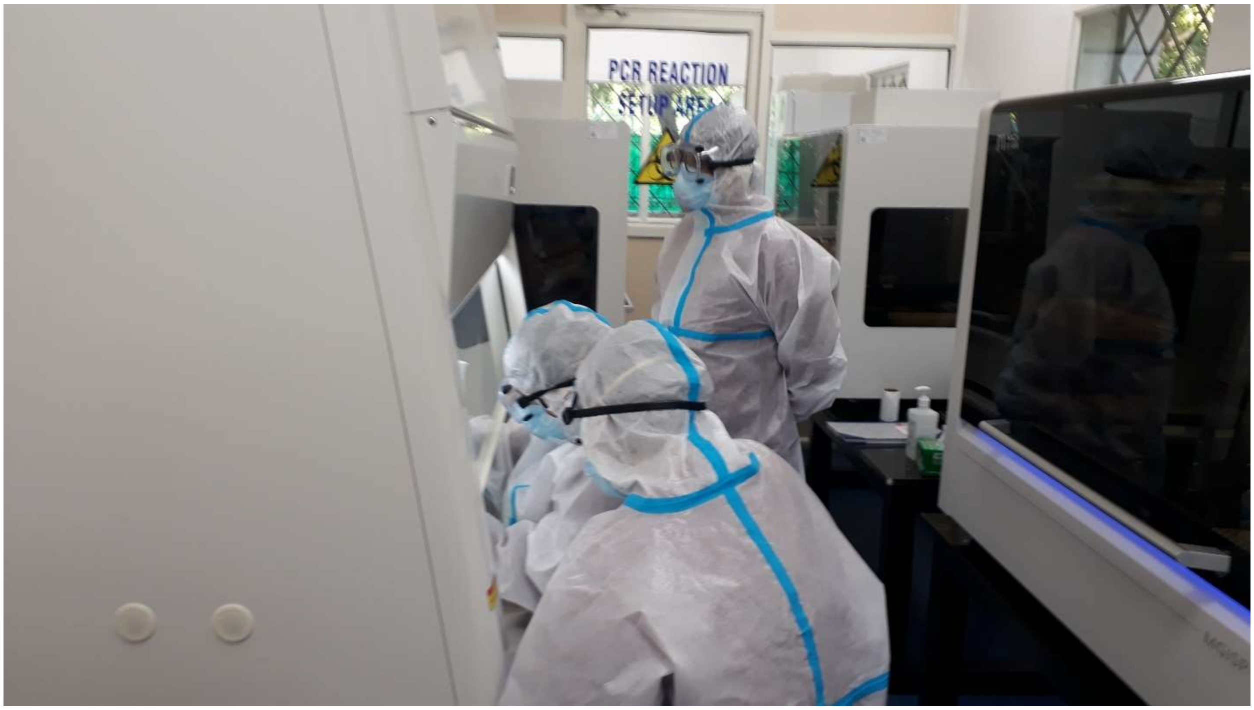

Figure 9: Staffs during RNA Extraction

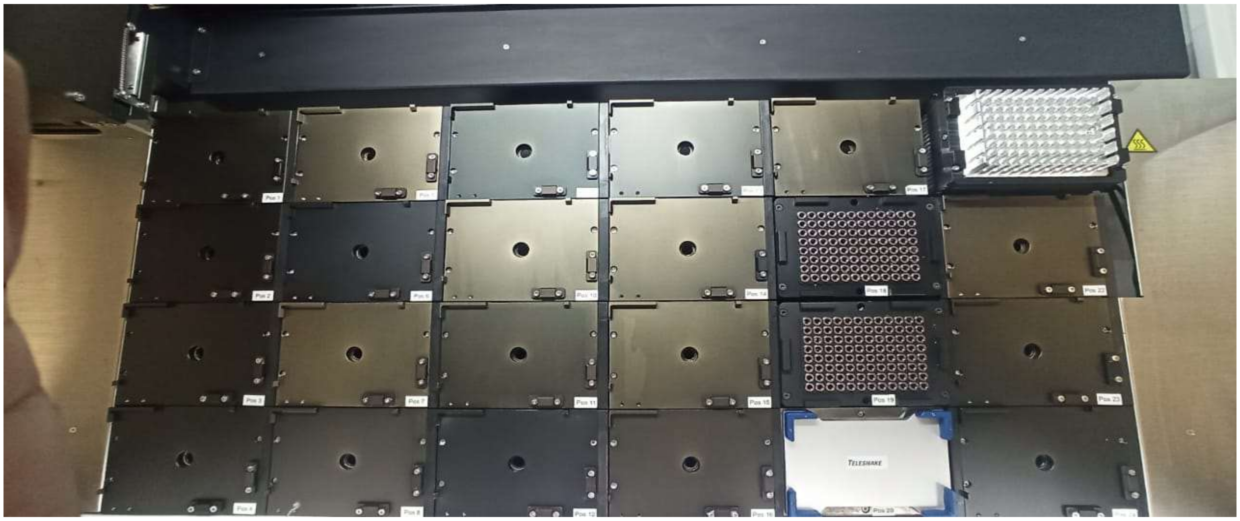

Figure 10: Positions 1 to 23 of MGISP 960 Automated RNA Extraction System

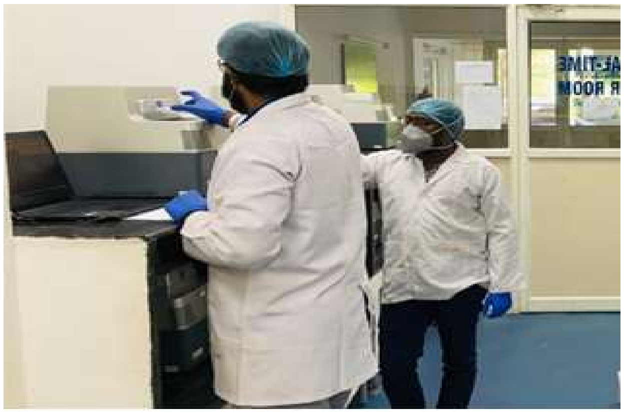

Figure 11: Staffs during PCR set up and analysis

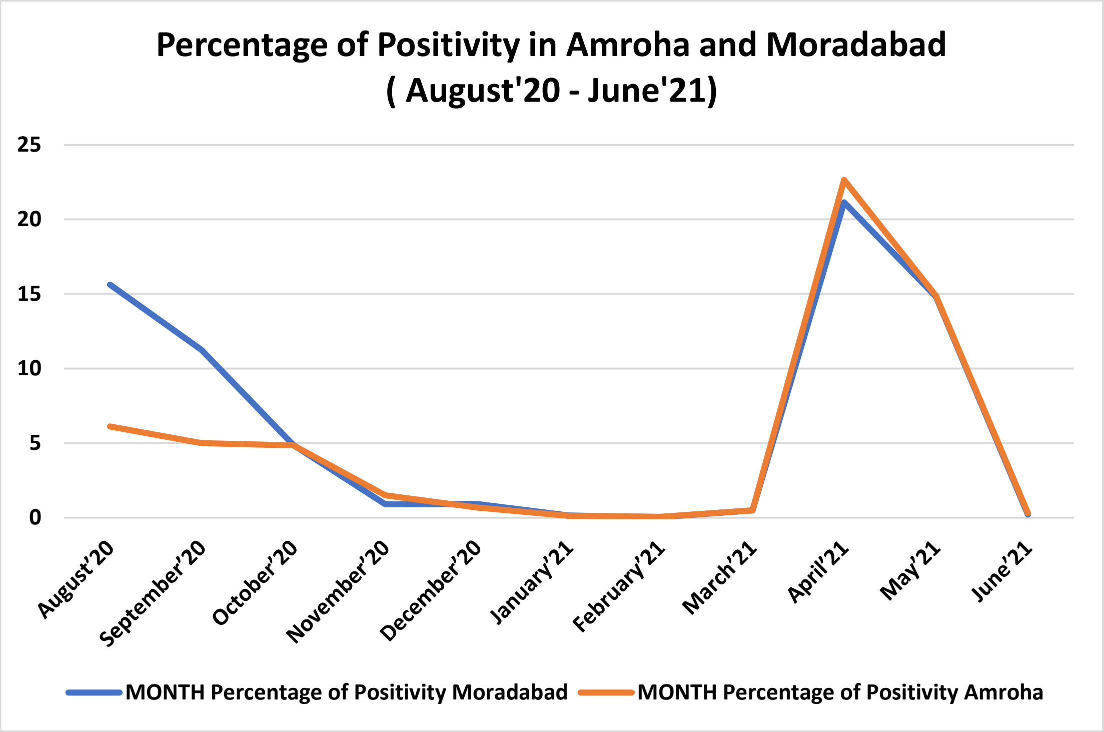

Figure 12: Percentage of Positivity in UP samples received from August'20 to June'21

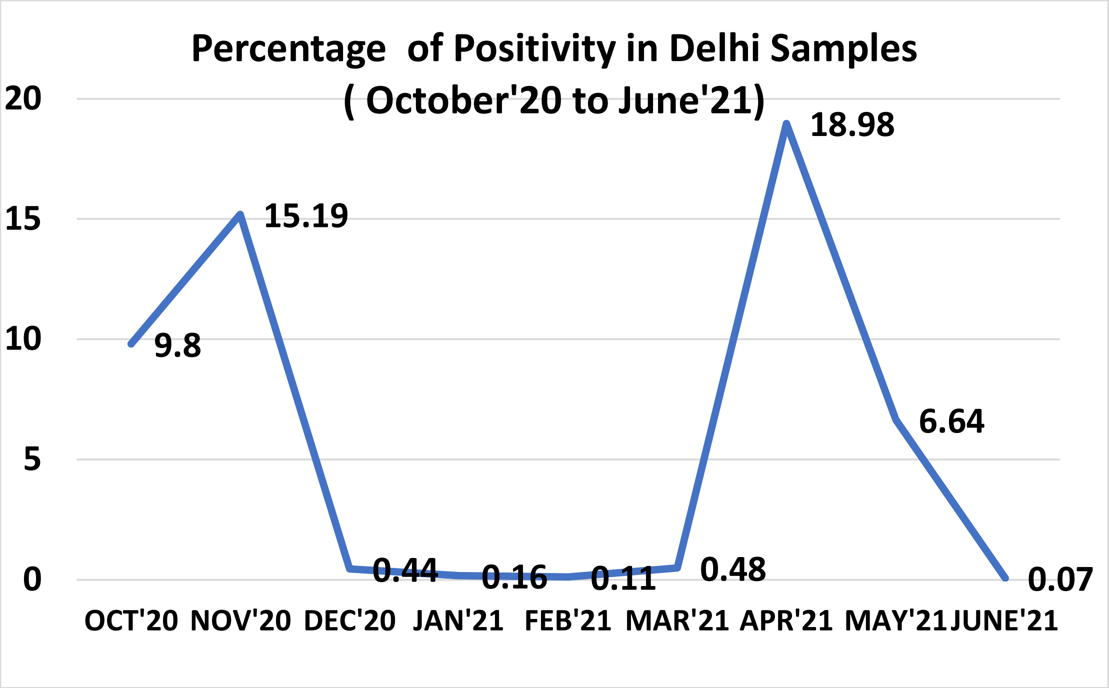

Figure 13: Percentage of Positivity in Delhi Samples received from October'20 to June'21.

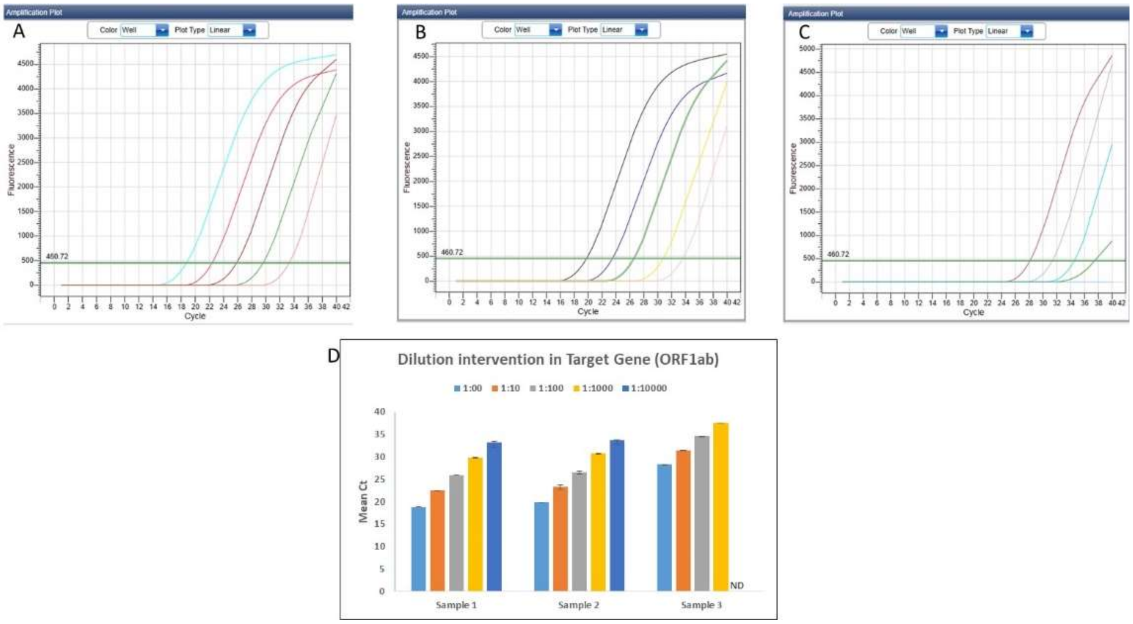

Figure 14: A) Amplification curve of sample 1 at different dilution. B) Amplification curve of sample 2 at different dilution. C) Amplification curve of sample 3 at different dilution. D) Graphs represented the mean Ct of 3 different samples with various dilution. ND= not detected.

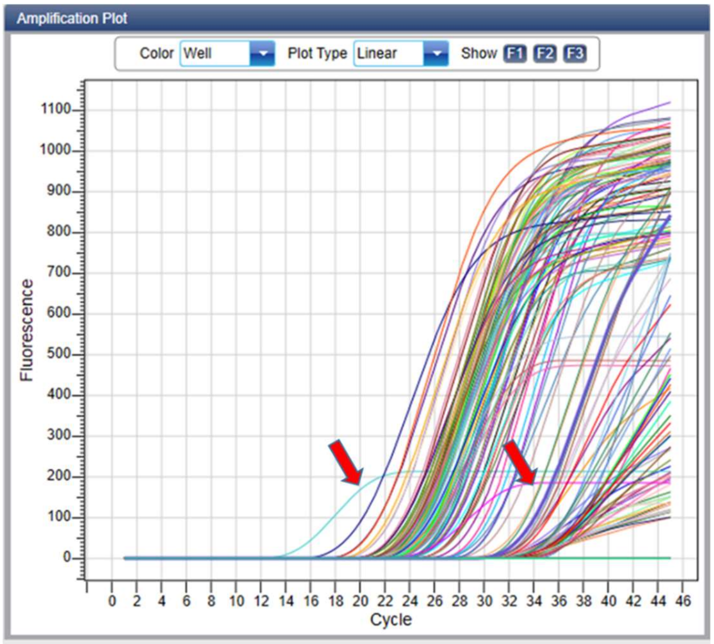

Figure 15: The image represents the false or early amplification caused due to bubble formation in well.

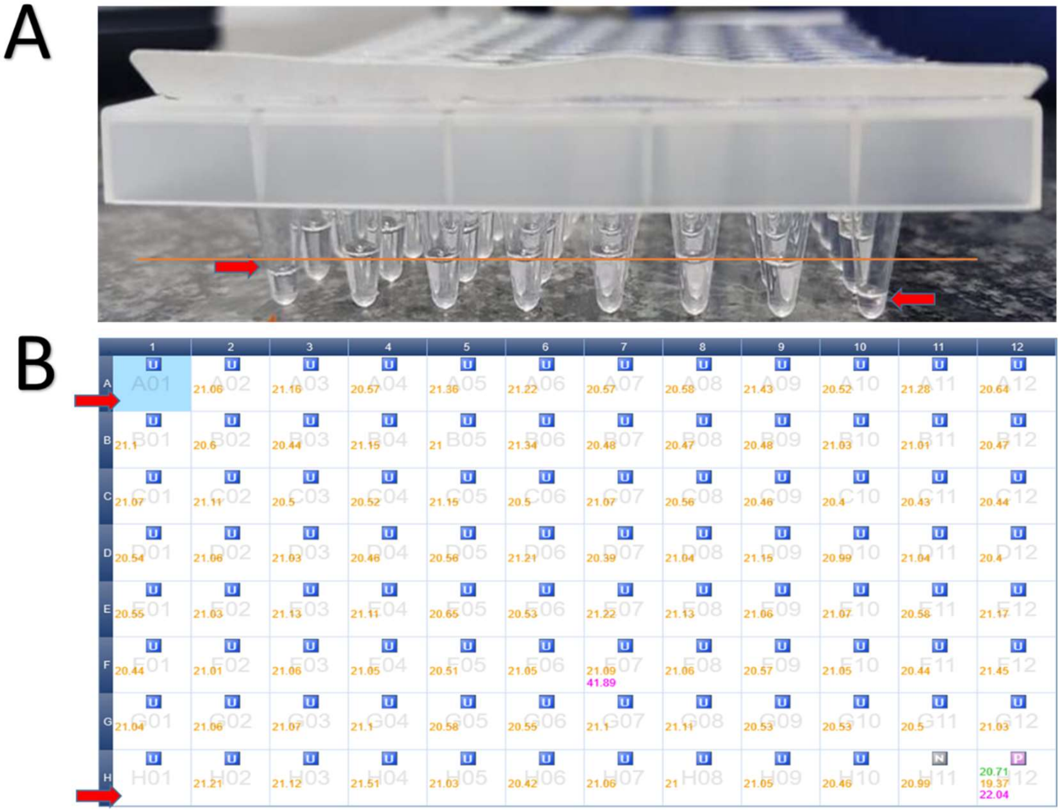

Figure 16: A) representative image of wells where samples get evaporated due to poor sealing. **B)** shows the representative results samples where no amplification found due to sample evaporation.

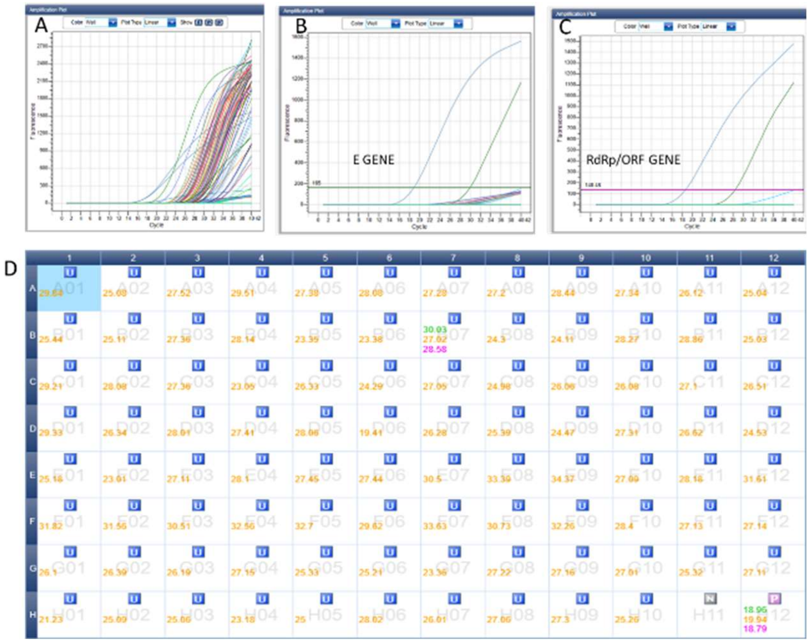

Figure 17: A) Representative amplification plot of a 96 well plate. **B)** The Base line for E gene was set manually to avoid background fluorescent. **C)** The Base line for RdRp/ORF gene was set manually to avoid background fluorescent. **D)** Representative results of a complete 96 well plate along with CT value of target gene and internal control. In this value in Orange colour represents Internal control, value in Pink colour represents ORF gene expression and Value in green colour represents E gene expression.

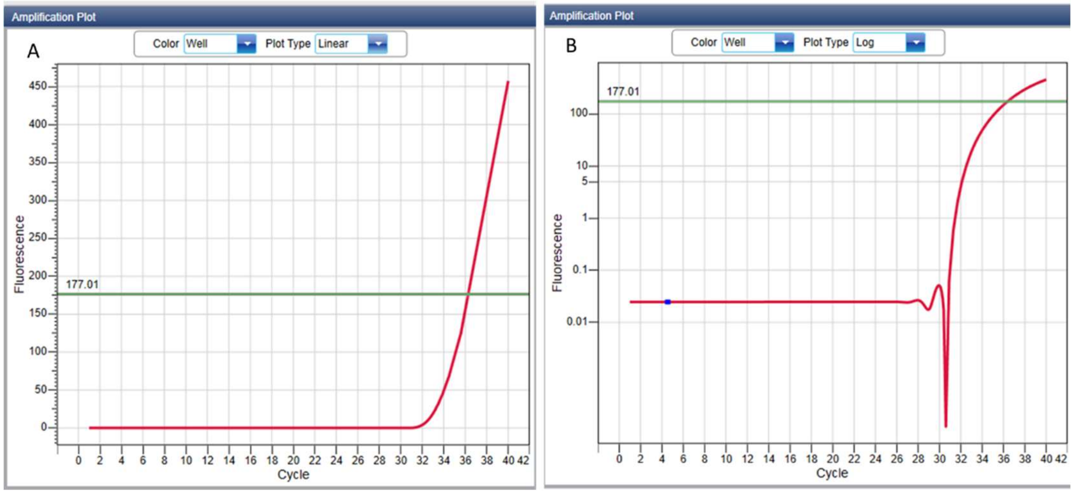

Figure 18: A) Image represents in linear scale. **B)** Image represents in log scale.

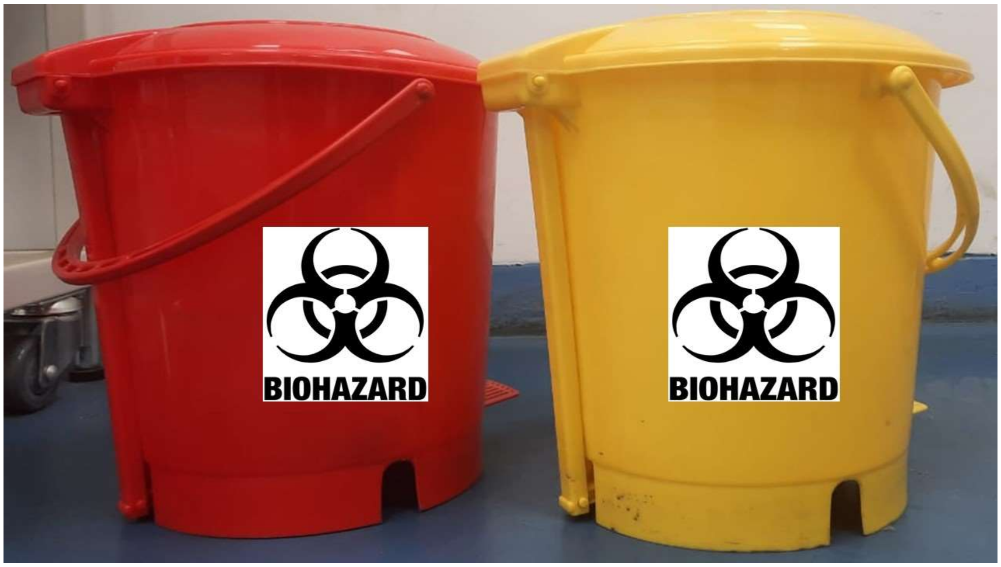

Figure 19: Different color-coded bin.

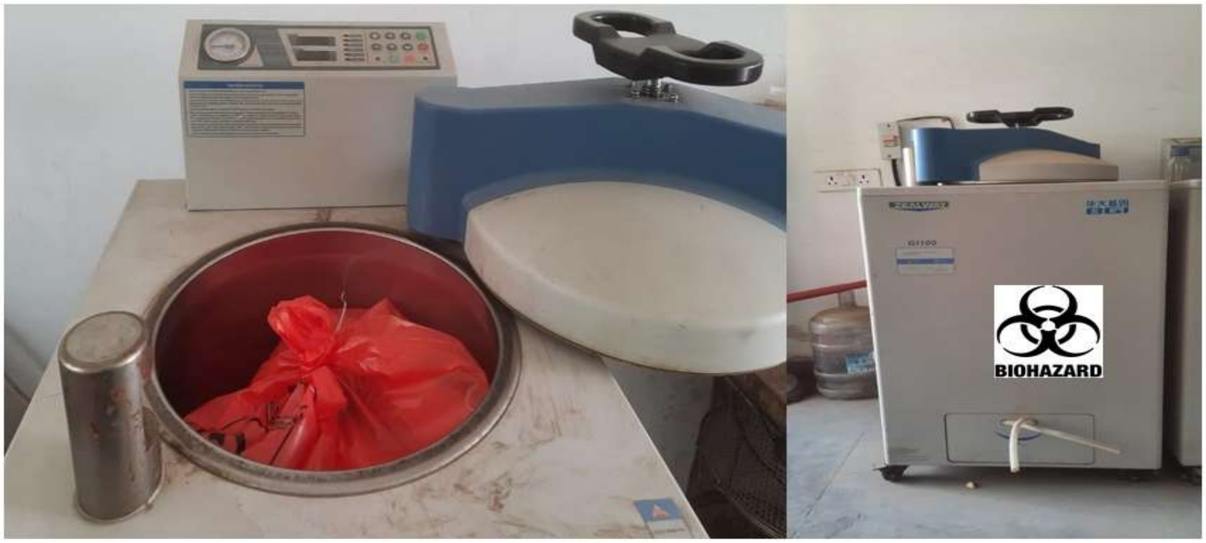

Figure 20: Autoclaving of Bio Hazard bags

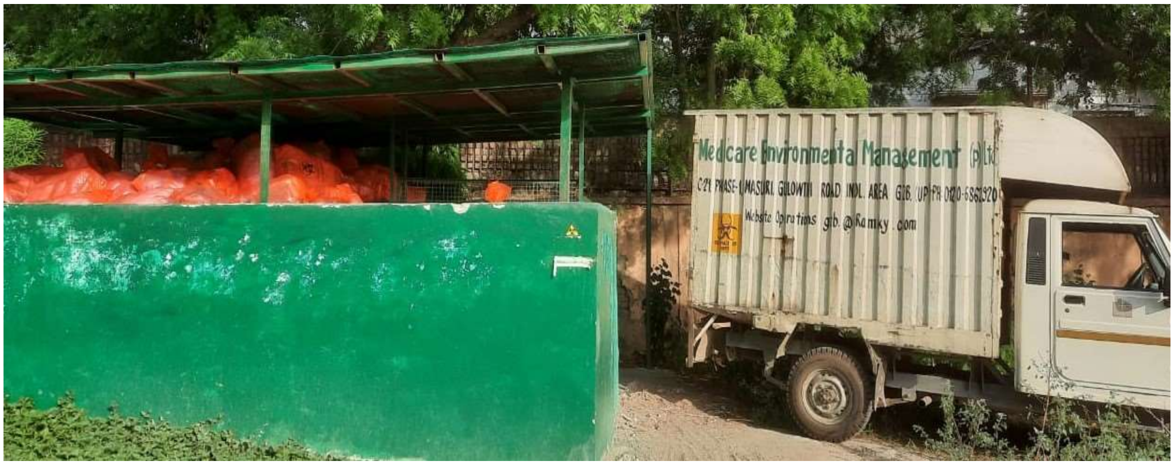

Figure 21: Collection of COVID-19 waste by CBWTF vehicle.
